# Mr. Taylor, on Digitalis

**Published:** 1808-12

**Authors:** Thomas Taylor

**Affiliations:** Surgeon. Harleston, Norfolk


### To the Editors of the Medical and Physical Journal.

"Books of Medicine, those monuments of Nature's frailty and Art's resources, when they treat of diseases, even the most trivial, would convince us that death was really at the door; but when they speak of the virtue of remedies, they place us again in marvellous security, as if we were immortal." MONTESQUIEU.

GENTLEMEN,

I Think your correspondent, Doctor Mossman, has indulged himself in a severity of censure, which is not altogether consonant with good breeding. Illiberal and unnecessary necessary reflections on the qualifications of another, do not tend to serve the cause of truth and science. I shall not retort upon Dr. Mossman, nor should I have even deemed it necessary to reply to him at all, but that he has drawn conclusions, which, I think, my paper did not warrant.

I certainly did not expect, when I transmitted to you my doubts, (and they were merely doubts) as to the usefulness of mercury in typhus fever, that they could have called forth so much angry and unprovoked attack from so many different quarters.

Too many of the pages of your respectable Journal, have already, I think, been devoted to this bootless controversy; I will, therefore, be as brief in my reply to Dr. Mossman as possible. For the sake of brevity then, since the Doctor has so gladly dropped the further consideration of mercury, I may limit myself to proving that the digitalis, the first article on his list, is falling into disrepute.

It is no great evidence of Dr. Mossman's attention to what he reads, if he infers, from my paper in the fourth number of your nineteeth volume, that I deny to the digitalis a powerful influence over arterial action. Its powers, in this respect, are unquestionable. Nor did I, as I recollect, *reprobate* the use and application of this vegetable; but I must be permitted still to doubt, whether its sanative powers be commensurate with the exaggerated and extravagant eulogies, as I conceive, of some, who have written on its medicinal properties; and, in expressing my doubts, I do not hold myself as "*unreasonably sceptical;*" nor, in exercising my own judgment, do I consider myself as "*unreasonably opiniative.*" Every medical man has a right to found his opinions on his experience, and I am ready to confess that, notwithstanding all Dr. Mossman has now, or on a former occasion, so vauntingly and confidently said on the subject, the powers of the digitalis have appeared to me much more limited than he would have me believe them.

I much question whether Heberden, whose book is indisputably a work of sterling merit, meant to express more than this, when he said: "Enervant omnia, quæ dicuntur narcotica, tum maxime herba digitalis."

I insinuated that the digitalis, with some other remedies, which I named, seemed to be destined to run the course that remedies usually do, when unreasonably extolled: i. e. to have their period of temporary celebrity and subsequent neglect. And I might here, perhaps, mention the cicuta cicuta as an example; a medicine which, not many years since, was administered with high expectation of curing cancerous disorders; and though it continues to be employed by some in those affections, yet, I imagine that it does not now hold the place in the opinions of medical practitioners, in general, that it once did.

Doctor Mossman refers me to a host of modern authors, in support of his opinion of the digitalis. Let us hear what they say; and then judge of Dr. Mossman's claim to candour and accuracy.

According to Dr. Hamilton, we know but little of what was done with the digitalis from the time of Fuchius down to Withering. That little, however, clearly evinces that the same discrepance of opinion respecting its properties and effects, which now exists, obtained then, and that it finally sunk into disuse.

Dr. Hamilton also quotes Sir George Baker as saying, that after the digitalis had been, in his time, esteemed a powerful remedy, it was, at another period, utterly rejected, as a plant, totâ substantiâ venenosâ.

Dr. Ferriar affirms, that it never suspended the appearances of *insanity* for a moment. He, moreover, gives in his second volume but a discouraging account of this vegetable; and, in his third volume, it is not even noticed! And yet, Dr. Mossman is absurd enough to refer me to this very work for proof of the marvellous effects of this plant!

Dr. Currie calls the digitalis a sedative poison, and condemns its use in *hydropic cases*.

Dr. Darwin, in the second volume of his Zoonomia, evidently begins to more than doubt of the beneficial properties of this medicine.

Dr. Mease, of Philadelphia, notices the disrepute into which the digitalis has fallen, in the following words. "The Foxglove is a medicine, which for some time stood high in the list of the Materia Medica, but for various reasons, appears to have in some measure lost the character once formed of it."

Dr. Beddoes is directly in opposition to Dr. Mossman respecting the use of digitalis in *pleurisy*. "If," "says Dr. B. "any one should be mad or wicked enough to forego the so certain resource of the lancet, in favour of the *sedative* virtue of the digitalis alone, he would, I apprehend, increase the disorder," &c.

Dr. Hamilton too joins in *reprobation* of the digitalis in *pneumonia*, and asserts that he has been "too often disappointed pointed in his hopes of sparing the further loss of blood, in inflammatory affections, and especially in pneumonia, by the agency of the digitalis; not to be satisfied, that, however respectable the authors by whom it is supported, the opinion of the powers of this remedy over inflammation is incorrect."

Does Dr. Mossman then expect me to pay more attention to his prayers and supplications in favour of the digitalis, and on the score of humanity too! than to the salutary warning of Dr. Beddoes, and the respectable authority of Dr. Hamilton? My experience of the digitalis in pneumonia, would determine me entirely to coincide with the respectable authorities just quoted, though, by so doing, I should hazard Dr. Mossman's resentment, and run the risk of being denounced as a monster for my inhumanity. Indeed, I have never found that "*the digitalis possesses powers approximating to specific*," in this disorder.

Dr. Hamilton adds, at the close of his Medical History of the Digitalis: "From this period little has been written on the subject of the digitalis, and the remaining part of the medical history of this plant can record little more than the instances in which it experienced almost total neglect. The periodical Journals have long ceased to exhibit the high wrought encomiums upon it, that a few years since so amply contributed to swell their pages; and the late authors who have written on the disorders, in which the beneficial agency of the digitalis was supposed most conspicuous, have not even mentioned it!"

Now what will Dr. Mossman say in answer to these passages, most of them cited from those very works to which he has so triumphantly referred. I should be sorry, however, to drive him (for he is a man of fine feelings on this subject) to despair; I must therefore add, by way of consolation, that, unfortunately, a great many consumptive patients are met with in this district, and that the digitalis has been usually given, aye, *and in the very form and dose* (matter, I hope, of pleasing astonishment to the Doctor) *as recommended by him, precisely as if we had been previously so instructed by his last communication.* And it has been so administered, because, as the Doctor has it, it is *a fashionable medicine,* besides, præstat certe anceps remedium experiri quam nullum.

The foregoing citations and remarks have already extended much beyond the limits which I had, at the outset, prescribed to myself; I shall, therefore, withhold my authorities authorities and reasons for the judgment I likewise pronounced on the uva ursi, till Doctor Mossman shall have made a suitable apology for the errors he has committed, and the rudeness of the language he thought proper to employ.

I am, &c.

THOMAS TAYLOR, Surgeon.

*Harleston, Norfolk, Nov. 12, 1808.*

P. S. Permit me to thank Dr. Mossman for his very ingenious exposition of the luminous diatriba of his newly adopted protegè. The text is worthy of the commentary!

